# Uncovering loggerhead (*Caretta caretta*) navigation strategy in the open ocean through the consideration of their diving behaviour

**DOI:** 10.1098/rsif.2023.0383

**Published:** 2023-12-13

**Authors:** Antoine Laforge, Philippe Gaspar, Anne Barat, Julien Temple Boyer, Tony Candela, Jérôme Bourjea, Stéphane Ciccione, Mayeul Dalleau, Katia Ballorain, Jonathan R. Monsinjon, Olivier Bousquet

**Affiliations:** ^1^ Laboratoire de l'Atmosphère et des Cyclones (UMR 8105 LACY), 15 avenue René Cassin, 97715 Saint-Denis, La Réunion, France; ^2^ Mercator Ocean International, 2 Av. de l'Aérodrome de Montaudran, 31400 Toulouse, France; ^3^ Upwell, Monterey, CA, USA; ^4^ MARBEC, Univ. Montpellier, CNRS, Ifremer, IRD, Avenue Jean Monnet, Sète 34200, France; ^5^ Kelonia, l'observatoire des tortues marines, 46 rue du Général de Gaulle, Saint Leu, La Réunion 97436, France; ^6^ Centre d’Étude et de Découverte des Tortues Marines (CEDTM), 6 Chemin Dubuisson 97436 Saint Leu, La Réunion, France; ^7^ French Research Institute for Exploitation of the Sea (IFREMER) - Indian Ocean Delegation (DOI), Le Port, La Réunion, France; ^8^ Institute for Coastal and Marine Research, Nelson Mandela University, Port-Elizabeth, South Africa

**Keywords:** loggerheads, satellite telemetry, swimming velocity, migration, open-ocean navigation

## Abstract

While scientists have been monitoring the movements and diving behaviour of sea turtles using Argos platform terminal transmitters for decades, the precise navigational mechanisms used by these animals remain an open question. Until now, active swimming motion has been derived from total motion by subtracting surface or subsurface modelled ocean currents, following the approximation of a quasi-two-dimensional surface layer migration. This study, based on tracking and diving data collected from 25 late-juvenile loggerhead turtles released from Reunion Island during their pre-reproductive migration, demonstrates the importance of considering the subsurface presence of the animals. Using a piecewise constant heading model, we investigate navigation strategy using daily time-at-depth distributions and three-dimensional currents to calculate swimming velocity. Our results are consistent with a map and compass strategy in which swimming movements follow straight courses at a stable swimming speed (approx. 0.5 m s^−1^), intermittently segmented by course corrections. This strategy, previously hypothesized for post-nesting green and hawksbill turtles, had never been observed in juvenile loggerheads. These results confirm a common open-ocean navigation mechanism across ages and species and highlight the importance of considering diving behaviour in most studies of sea turtle spatial ecology.

## 1. Introduction

The ability of migratory animals to return to a specific location with pinpoint accuracy from a great distance, sometimes after decades of absence, continues to puzzle scientists [1]. An emblematic example is the return of sea turtles to their birth location after decades of pelagic and neritic development, which often involves crossing entire oceans to reach a specific beach only a few kilometres wide. This general behaviour, known as natal philopatry [2], is particularly challenging in dynamic oceanic environments as drift usually prevents the animals from taking the most direct routes [3,4]. Understanding how evolution has shaped the navigational mechanisms of migratory animals is key for developing movement prediction models that can inform the spatial distribution of these vulnerable species and ultimately influence conservation policies.

Decades of scientific work have shed new light on this remarkable feat, and numerous experiments have already demonstrated the ability of migrating animals such as birds, salmon, sea turtles and many other taxa, to rely on the Earth's magnetic field for navigation [5–10]. Although the clear understanding of the exact navigation mechanisms involved during the migration of these animals is still an active research topic, its potential at providing sufficient orientation cues to reach a far target has been demonstrated in many studies [11–14]. Among the numerous proposed navigation strategies, the most prolific is the one known as the map and compass strategy [15]. This approach consists in locating relatively to a distant target (the map) to define an approximate heading to be followed in a straight-line manner (the compass). Recent findings seem to demonstrate the potential of this navigation strategy for sea turtles aiming at a wide continental target [16–18], or travelling toward isolated islands [19,20] during their open-ocean migrations.

Unravelling the navigational strategy of marine animals requires isolating the intended active movement from the transport imposed by the environment at the location of the individuals [21]. This requires precise knowledge of both the position of the individual and the dynamics of the surrounding fluid. However, the complexity of ocean dynamics and our ability to accurately monitor the spatial behaviour of sea turtles in their three-dimensional environment can lead to significant errors in estimating their intended movement. Recent advances in animal tracking, which allow accurate reconstruction of three-dimensional movements of animals [22], and improvements in physical oceanographic modelling (e.g. [23]), are nonetheless expanding the possibilities for understanding the navigational strategies of sea turtles.

As part of the Sea Turtles for Ocean Research and Monitoring (STORM) programme [24,25], 32 late-juvenile loggerhead turtles rescued from accidental longline bycatch at the Sea Turtle Observatory of Reunion Island (Kelonia) were tagged with Argos platform terminal transmitters (PTTs) equipped with time-depth recorders (TDRs) upon release into the southwest Indian Ocean. Of these 32 individuals, 25 migrated north, crossing the western Indian Ocean to recruit in the neritic habitats of the Arabian Peninsula. This migration route has been identified as a major corridor for late-juvenile loggerheads from the northwestern Indian Ocean, which are thought to undertake their first reproductive migration after years of development in the southern Indian Ocean [26,27]. As the destination of these inexperienced first-time migrants is roughly known, the open-ocean transects recorded during their journey provide a unique insight into their goal-directed navigation strategy.

This paper presents a new approach to estimate active swimming velocity using daily time-at-depth (TAD) distributions in combination with three-dimensional ocean model output. The novel three-dimensional swimming velocity is compared with the commonly used two-dimensional approach in terms of normality, relative error and straightness to evaluate the relevance of the new method. We apply a segmentation algorithm on the swimming heading using this new approach to investigate the hypothesis of a piecewise constant heading navigation strategy. Finally, the following questions are discussed:

(1) When is it important to choose a three-dimensional approach for estimating swimming velocity in marine animals?
(2) What navigational strategy do juvenile loggerhead turtles rely on during their pre-reproductive migration?

## 2. Material and method

### 2.1. Turtles and tag deployment

#### 2.1.1. Tag deployment

Each year, approximately 20 late-juvenile loggerhead turtles are accidentally captured in the southwest Indian Ocean by French longliners fishing off the coast of Reunion Island [28]. Following a certified protocol described in [26], these injured turtles are returned to Kelonia for rehabilitation and later release into the ocean. Between January 2019 and April 2022, 32 of these rehabilitated late juveniles (average straight carapace length (SCL) = 67.6 ± 6 cm) were released from a beach adjacent to the Kelonia care centre (21.15° S, 55.28° E) as part of the STORM research programme [24,25]. All animals were fitted with Argos PTTs attached to their carapace, following the procedure described in [26]. Two types of PTTs, both containing environmental sensors and TDRs, were used: (i) the SPLASH10-344D-02 OCTAGON manufactured by Wildlife Computers (WC, *N* = 23) and (ii) the K2G 376E DIVE PU manufactured by LOTEK (*N* = 9). Tracking and TDR data were transmitted via the Argos satellite system and retrieved from tag manufacturers' web portals.

#### 2.1.2. Tracking data

**Figure 1.**
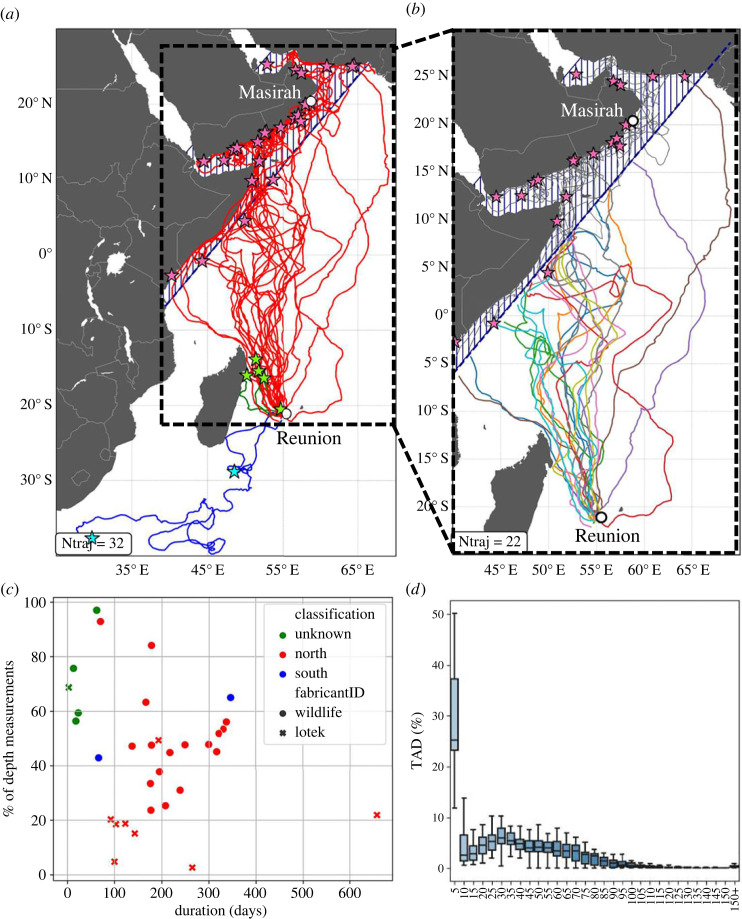
(*a*) Satellite tracks of the 32 late-juvenile loggerhead turtles released from Reunion Island between 2019 and 2022. Tracks are grouped into three categories: North (red), South (blue) and Unknown (green) based on their last recorded position (respectively, red, blue, and green stars). (*b*) Tracks of the 25 animals migrating north (group North) used in this study with their last recorded position (red stars). The blue dashed area in (*a*) and (*b*) indicates where all individuals from group North were considered to have ended the open-ocean portion of their track. (*c*) Relationship between tag life duration and the average percentage of depth measurements recovered for all 32 tracks (from table 1) classified by group (colour) and by tag manufacturer (dots for WC, crosses for LOTEK). (*d*) Inter-individual time-at-depth (TAD) distribution for the open-ocean transect of group North turtles (*N* = 25).

Of the 32 recorded tracks (table 1 and figure 1*a*), 25 animals migrated north towards the Arabian Peninsula (group North: 78%), two migrated south towards the subtropical front (group South: 6.2%) and five tags did not last long enough for their destination to be clearly identified (group Unknown: 15.6%). From a similar dataset, [26] observed the same dispersion pattern and figures, despite a higher proportion (28%) of short tracks classified as unknown (most likely due to less reliable tags available at the time of their study). Genetic studies of other late-juvenile loggerheads rehabilitated at Kelonia between 2013 and 2014 showed that 80% of the animals originated from the northwest Indian stock [27], with Masirah Island being the main rookery [29,30]. This strongly suggests that the 25 northward migrating late-juveniles tracked in the present study were undertaking their first pre-reproductive migration (first-time migrants), targeting neritic feeding areas close to their natal region [31] before reaching sexual maturity and possibly mate and reproduce. As the destination of the first group of individuals (*N* = 25) is roughly known, with Masirah Island being considered as their most likely destination, their trajectories were selected to investigate open-ocean navigation strategies (figure 1*b*). Although all the tags did not transmit all the way to the coast of the Arabian Peninsula (see pink stars in figure 1*b*), all the sea turtles of this group reached at least the coast of Somalia before transmission was lost, thus providing all their positions during open-ocean transects. As the navigational paradigm changes upon reaching the Somali coast (either from open-ocean to coast-oriented navigation or from pure migratory behaviour to foraging), movements in coastal areas (blue hatched area in figure 1*b*) are not investigated in this study.

**Table 1.** Identification number, PTT number, group, tag manufacturer, deployment date, last transmission date, SCL (cm), weight (kg), track duration (days) and the average percentage of depth data (as defined in §2.1.3) for the 32 late-juvenile loggerheads released from Reunion Island sorted by order of group and deployment date. The trajectories selected for the study are provided in the 25 first rows.

ID	PTT	name	group	tag manufacturer	deployment date	last date	SCL (cm)	weight (kg)	duration (days)	Ave. % depth
1	65712	Brice	North	WC	2 Apr 2019	7 Nov 2019	75.0	66.4	219	44.2
2	65722	Samson	North	WC	15 Feb 2019	31 Aug 2019	69.0	59.3	197	37.3
3	178936	Tina	North	LOTEK	21 May 2019	9 Mar 2021	66.5	47.8	658	22.8
4	202350	Celou	North	WC	8 Dec 2020	13 Nov 2021	67.0	51.6	340	56.0
5	202487	Curieuse	North	WC	3 Nov 2020	11 July 2021	74.0	74.0	250	46.1
6	197625	India	North	LOTEK	26 Feb 2020	18 Nov 2020	62.0	42.6	266	2.8
7	202349	Petit Toussaint	North	WC	19 Nov 2020	4 Oct 2021	68.7	55.4	319	43.7
8	197624	Tom	North	LOTEK	19 Feb 2020	3 June 2020	66.9	57.4	105	4.8
9	223934	Amayah	North	WC	28 Oct 2021	16 Mar 2022	57.1	31.3	139	45.6
10	202352	Cassandre	North	WC	5 Jan 2021	4 Dec 2021	69.0	59.4	333	54.7
11	202354	Cassie	North	WC	23 Feb 2021	20 Dec 2021	63.0	48.5	300	51.2
12	210001	Davina	North	WC	13 Apr 2021	9 Dec 2021	67.0	58.9	240	30.7
13	202353	Germaine2	North	WC	19 Jan 2021	8 Dec 2021	65.0	45.0	323	53.6
14	205575	Isabelle	North	LOTEK	18 May 2021	28 Nov 2021	69.5	62.1	194	50.2
15	210000	Mona	North	WC	29 Jan 2021	29 July 2021	69.0	63.2	181	21.9
16	223935	Oulanga	North	WC	25 Nov 2021	23 June 2022	65.0	49.2	210	23.7
17	224025	Arthur	North	WC	12 Jan 2022	11 July 2022	67.0	51.4	180	46.4
18	224008	Hercule	North	WC	31 Mar 2022	15 Sep 2022	68.0	50.2	168	65.5
19	226009	Jacqueline	North	LOTEK	7 Mar 2022	12 July 2022	72.5	62.3	127	17.4
20	224014	Jean-Louis	North	WC	14 Jan 2022	12 July 2022	66.0	53.6	179	65.3
21	224027	Lyvan	North	WC	15 Jan 2022	12 July 2022	74.5	65.5	178	34.6
22	226011	Maina	North	LOTEK	31 Mar 2022	2 July 2022	69.2	57.5	93	18.5
23	226008	Margot	North	LOTEK	18 Feb 2022	12 July 2022	64.0	40.1	144	14.8
24	226010	Oriane	North	LOTEK	29 Mar 2022	12 July 2022	62.5	45.3	105	17.2
25	224026	Tiago	North	WC	14 Jan 2022	22 May 2022	61.0	43.5	128	67.2
26	65711	Fifi	South	WC	7 Oct 2019	14 Dec 2019	88.0	115.2	68	41.7
27	202351	Oscar	South	WC	14 Dec 2020	28 Nov 2021	53.0	26.8	349	65.3
28	657110	Ilona	unknown	WC	9 Jan 2019	16 Mar 2019	72.0	69.2	66	96.3
29	178937	Katty2	unknown	LOTEK	2 May 2019	6 May 2019	63.5	45.8	4	50.7
30	180908	Tikaf	unknown	WC	30 Sep 2019	19 Oct 2019	68.4	58.4	19	53.7
31	180909	Sylvia	unknown	WC	17 June 2021	3 July 2021	71.0	71.3	16	47.4
32	224024	Camille	unknown	WC	18 Feb 2022	14 Mar 2022	62.0	43.1	24	55.8

#### 2.1.3. Diving data

In addition to tracking data, all tags also provided time-series records of temperature and depth at a sampling rate of 5 min and a resolution of 0.05°C and 0.5 m, respectively. The performance of these tags is evaluated in figure 1*c* in terms of the daily average of recovered 5 min depth readings (*N*_5min_/(12 × 24) in percentage) as a function of the tag lifetime (in days). The average tag lifetime is 191 (±131) days, with equivalent performance for both tag models (192 days for WC tags and 188 days for LOTEK tags). The percentage of recovered depth measurements has a global mean of 42%, with a standard deviation of 20%. The mean is markedly higher in WC tags (49%) than in LOTEK tags (22%) (figure 1*c*).

### 2.2. Three-dimensional oceanographic data

**Figure 2.**
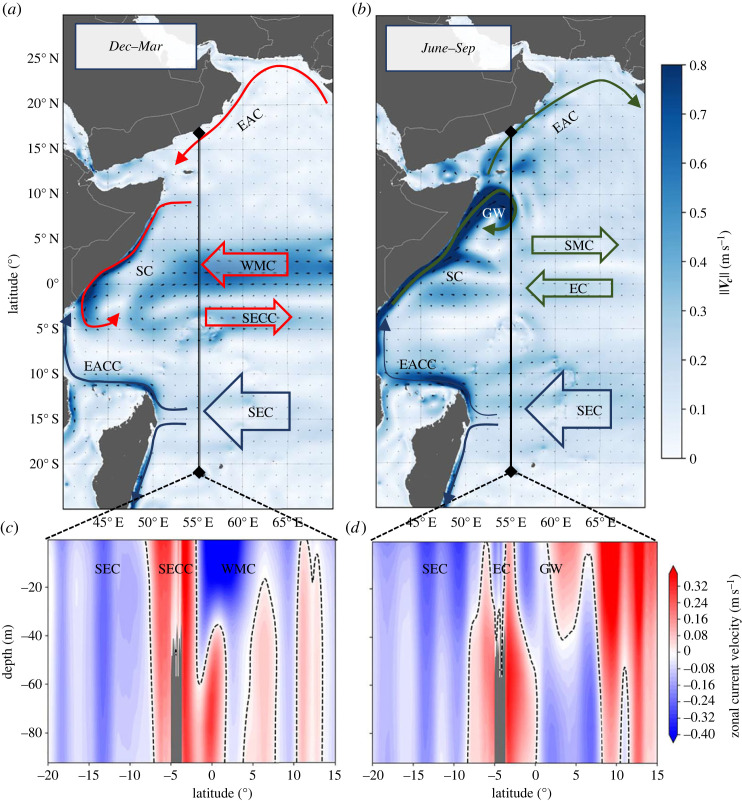
West Indian Ocean current circulation captured by Mercator Ocean's operational model PSY4. (*a*,*b*) Surface current climatology (2008–2021) in December–March (winter monsoon) and June–September (summer monsoon). (*c*,*d*) Associated vertical structure of the zonal (east–west) currents from the surface to 100 m depth along the 55° E meridian. Displayed currents are the SEC, South equatorial current; EACC, East African Coastal current; SECC, South equatorial counter current; SC, Somali current; WMC, Winter monsoon current; SMC, Summer monsoon current; EC, Equatorial current; GW, Great Whirl; EAC, East Arabian current.

Current velocity *V_c_* is retrieved from the PSY4 operational oceanographic model developed at Mercator Ocean International [23] and operated in near real-time by the Copernicus Marine Environment Monitoring Service [32]. Daily current velocity fields are provided with 1/12° horizontal resolution (approx. 9.3 km at the equator) over 50 vertical levels (0.49–5727 m), with spacing increasing with depth from 1 m at the surface to 450 m at the bottom. The model, which assimilates satellite measurements and *in situ* temperature/salinity vertical profile measurements, is able to model the seasonal variability (the monsoon circulation) as well as the vertical structure of the Western Indian Ocean (WIO). At the surface and between December to March, the northern part of the Somali current (SC) flows southward, feeding the South Equatorial counter current (SECC), while a strong zonal Winter monsoon current (WMC) flows westward (figure 2*a*) in agreement with [33]. From June to September, the SC flows northwards, fed by the East African Coastal current (EACC), while the equatorial Summer monsoon current (SMC) flows eastwards (figure 2*b*). In the third dimension, PSY4 is able to model the complex vertical structure of the WIO. Figure 2*c* shows the zonal current inversion across 55° E during the winter months (December–March). The WMC also presents a current inversion with currents flowing westward below 40 m at the equator and below 20 m around 5° N. During the summer months (June–September, figure 2*d*), the easterly branch of the Great Whirlpool (GW) shows a vertical inversion of its flow at 40 m depth.

Bathymetry was obtained from the General Bathymetric Chart of the Oceans product (GEBCO) [34], a publicly available chart of bathymetric data gridded on a 15 arc-second interval grid (460 m at the equator).

### 2.3. Swimming velocity

The trajectory of sea turtles in the ocean results from a combination of the active swimming motion and of the drift induced by the ocean currents at the position and depth of the individual [21]. The active swimming velocity ***V_s_*** at position *x*(*t*) and depth *d*(*t*) is derived from the ground velocity ***V_g_*** and the current velocity ***V_c_*** (equation (2.1)). Note that velocity vectors ***V*_{s,g,c}_** are shown in bold to simplify the notation.

2.1 $$V_{s}(t)=\;V_{g}(t)-V_{c}(x(t),d(t),t)$$

Since PSY4 provides daily averages of the current velocity fields centred at *t*_mid_ = 12.00 UTC, hereinafter referred to as $\hat{V_{c}}$, the ground displacement from which ***V_g_*** is derived, had to be estimated over daily time steps. To do this, Argos locations are interpolated at 00.00 and 12.00 UTC using the *aniMotum* R package [35]*.* From there, the daily ground velocity $\hat{V_{g}}$ was estimated at *x*(*t*_mid_) from the distance travelled between positions *x*(*t*_mid_ − 12 h) and *x*(*t*_mid_ + 12 h).

The daily current velocity $\hat{V_{c}}$ is estimated at *x*(*t*_mid_) by inverse distance weighting (IDW) interpolation of the four adjacent tiles. $\hat{V_{c}}$ must necessarily consider the depths at which the animal evolves over these 24 h windows. Sea turtles are known to occupy the upper layer of the water column when migrating through pelagic areas [36–39]. For this reason, surface or near-surface current values have been systematically used as a simplification to estimate swimming velocity, especially when dive data or subsurface current data were not available [8,17,20,21,40,41]. However, this approximation can be biased when: (i) the current velocity field is not uniform along the vertical axis in the study area, and (ii) the daily individual time-at-depth (TAD) distributions show significant residence time in subsurface layers.

In the context of our study, both conditions are found. The WIOs show seasonal vertical shear at depths where the studied individuals spend at least half of their time during migration. Figure 1*d*, which shows the inter-individual TAD integrated over the open-ocean transects, shows an average time ratio of about half/half above and below the 40 m layer, which is the maximum depth where current inversions were found (figure 2*c,d*). Therefore, the upper-layer migration approximation is questionable here and motivates the need to consider both the TAD and the vertical structure of the ocean currents to accurately estimate the currents encountered and consequently the swimming velocity.

To assess on the benefits of using a three-dimensional approach relatively to a two-dimensional surface approach, two current velocities $\hat{V_{c}}$ are considered here:

(a) ${\hat{V}}_{c_{0}}=\hat{V_{c}}(x(t_{\mathrm{mid}}),d=0,t_{\mathrm{mid}})$, which uses the upper-layer migration approximation and applies surface currents to calculate the swimming velocity, hereinafter referred to as ${\hat{V_{s}}}_{0}$,
(b) ${\hat{V}}_{c_{\left[\mathrm{TAD}\right]}}$ (equation (2.2)), which considers all vertical layers d(*t*) visited by the animal during these 24 h windows to estimate a more realistic encountered swimming velocity, hereinafter referred to as ${\hat{V}}_{s_{\left[\mathrm{TAD}\right]}}$
  2.2 $${\hat{V}}_{c_{\left[\mathrm{TAD}\right]}}=\underset{n}{\sum }{\mathrm{TAD}}_{n}\times {\hat{V}}_{c_{n}}$$
  where TAD*_n_* is the normalized distribution of the proportion of time per days (ranging from 0 to 1) spent at depth bin *n* (table 2). It is estimated from the daily time-series of depth records with depth bins chosen to match the 30 first vertical layers of the PSY4 model, thus allowing for direct correspondence between TAD*_n_* and ${\hat{V}}_{c_{n}}$.

The calculation of ${\hat{V}}_{c_{\left[\mathrm{TAD}\right]}}$ following equation (2.2) requires the knowledge of all depth records, while we know from §2.1.3 that less than half of the depth records are available. Fortunately, tag no. 28 (Ilona) has been physically recovered so that the full time series of depth records along with high-frequency sampled TAD histograms are available. Analysis of these data shows that a representative daily TAD distribution can be obtained from only 16% of the 5 min daily depth records for both tag manufacturers (see electronic supplementary material, S1). Using this criterion, we were able to estimate ${\hat{V}}_{c_{\left[\mathrm{TAD}\right]}}$ for 81% (*N* = 1821) of the daily positions of the group North trajectories.

**Table 2.** The 30 first vertical layers of PSY4 model which define the bins *d* of the TAD distribution. The depth, provided in metres, defines the lower boundary of each bin.

**bin (*n*)**	**0**	**1**	**2**	**3**	**4**	**5**	**6**	**7**	**8**	**9**	**10**	**11**	**12**	**13**	**14**
depth (*d*)	0	0.5	1.5	2.6	3.8	5.1	6.4	7.9	9.6	11.4	13.5	15.8	18.5	21.6	25.2
**bin (*n*)**	**15**	**16**	**17**	**18**	**19**	**20**	**21**	**22**	**23**	**24**	**25**	**26**	**27**	**28**	**29**
depth (*d*)	29.4	34.4	40.3	47.4	55.8	65.8	77.8	92.3	109.7	130.7	155.8	186.1	222.5	266.0	318.1

### 2.4. Current-corrected tracks

The open-ocean navigation rules can be difficult to unfold solely from the Argos satellite reconstructed track (hereafter referred to as the *real track*: *X_g_*) as ocean current variability masks part of the swimming activity. Therefore, it is common to evaluate the animal's navigation strategy by analyses of the *current-corrected track*. This fictive track, calculated by integration of $\hat{V_{s}}$, represents the movement of the animal in a motionless ocean [21,40,41].

The calculation of the *current-corrected track* necessarily requires gapless swimming velocity estimates along the trajectory. However, as shown in the previous section, ${\hat{V}}_{s_{\left[\mathrm{TAD}\right]}}$ contains 19% gaps over the entire dataset. Therefore, to estimate reliable *current-corrected tracks*, we discard tracks or track sections that (i) contain gaps longer than 48 h to limit the gap length, and (ii) contain more than 20% of gaps over the entire track to ensure sufficient data density. Based on these restrictions (see electronic supplementary material, S2 for details): India (no. 6), Tom (no. 8), Mona (no. 15), Jacqueline (no. 19), Margot (no. 23) and Oriane (no. 24) are discarded. Petit Toussaint (no. 7), Davina (no. 12), and Tiago (no. 25) are truncated to remove gaps longer than 48 h that occur at the beginning or end of their open-ocean transect. These restrictions ensure that enough valid data are available to estimate the swimming velocity in the remaining gaps (98 out of 1684 daily positions) by linear interpolation between adjacent current velocity. Note that these constraints apply only to when the *current-corrected track* is investigated (§3.3), when the need for continuity is not required, the total of 1821 valid daily positions is used.

In the present study, the swimming velocity vectors are used with their polar coordinates (‖V(t)‖,θ(t)), where ‖V(t)‖ is the velocity norm in m s^−1^, hereafter referred to as the speed, and *θ*(*t*) is the direction of movement with respect to the north, hereafter referred to as the heading. The tracks corresponding to the velocities studied: ${\hat{V}}_{s_{\left[TAD\right]}}$, ${\hat{V}}_{s_{0}}$, ${\hat{V}}_{g}$ are denoted respectively by *X*_[*TAD*]_, *X*_0_, *X_g_*.

### 2.5. Course correction breakpoints

To investigate the navigation strategy of sea turtles in the open ocean, we assess whether turtles sequentially follow different headings, i.e. whether trajectories are well approximated by a sequence of nearly straight legs. This is done by applying a standard breakpoint detection algorithm to the heading time series *θ*_[*TAD*]_(*t*). For any time series, breakpoint detection consists of finding the optimal segmentation by minimizing a cost function [42,43]. The cost function (which can be considered as a measure of the homogeneity of each segment) is here chosen as a non-parametric kernel-based detection model with a Gaussian kernel (cf. [44,45]). The binary segmentation algorithm available in the *rupture* python package [46] is used to search for the breakpoints. The complexity of finding the optimal number of breakpoints lies in minimizing the total cost function using a limited number of breakpoints. To that end, we follow the approach of [47], revised by [48], to detect only clear course changes and to minimize the number of false positives. We plot the total cost (Cost) for a set of change points (*Nbkps*) and select the ‘elbow’ point where − *dCost*/*dNbkps* is maximal, indicating optimal segmentation. This method effectively detects consistent heading changes with minimal breakpoints (see results in figure 5 and electronic supplementary material, S3, figure S3.1, table S3). The main limitation of this approach is the inability to select the no change point as the best solution.

To evaluate the performance of the segmentation, the root mean square error (RMSE) is calculated for each segment. Residual errors in the current correction or changes in behaviour along the way may prevent the model from finding a constant heading, resulting in high RMSE for these segments. Monsinjon *et al*. [49] using a hidden Markov model (HMM) on a similar dataset (*n* = 12 common tracks) showed that approximately 23% of the daily position could be associated with a ‘foraging' state. By comparing the proportion of daily positions marked as ‘foraging' within each segment with the RMSE, we estimated that a threshold of 30° of RMSE was an acceptable compromise to remove segments (6 out of 47) where the large heading variability indicated foraging rather than directed swimming (see electronic supplementary material, S3, figure S3.2). Note that breakpoints adjacent to an invalid segment are retained in the statistics, as they represent a change in global heading, even if the nature of the invalid segment is not fully understood.

For the valid segments (RMSE < 30°) the RMSE and the straightness index (S) for *X*_[*TAD*]_, *X*_0_ and *X_g_* are calculated. The objective is to evaluate and quantify the benefits of (i) correcting from surface currents (from *X_g_* to *X*_0_) and of (ii) applying the TAD methodology (from *X*_[*TAD*]_ to *X*_0_) to uncover the piecewise constant heading navigation strategy. As both the RMSE and the straightness index depend on the segment size, it is convenient here to make pairwise comparisons between the same segments.

## 3. Results

### 3.1. Impact of the current vertical shear

**Figure 3.**
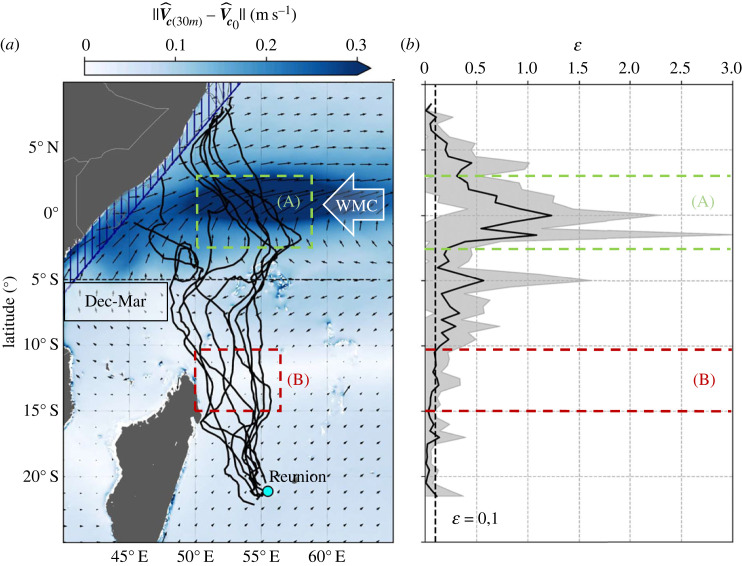
(*a*) Tracks that cross the 5° S parallel between December and March (*N* = 9) atop the norm of the current speed climatology (2008–2021) difference between 30 m of depth and the surface during the same period. Regions (A) and (B), respectively, define regions of strong and weak vertical shear. (*b*) the average relative error (*ε*) of ${\hat{V_{s}}}_{0}$ w.r.t. $\;{\hat{V_{s}}}_{\left[\mathrm{TAD}\right]}$ as a function of latitude bounded by the interval [mean − s.d.; mean + s.d.].

To assess the impact of the variation in vertical current shear on the swimming velocity vector, the tracks of the juvenile loggerheads crossing oceanic areas of both low and high vertical current shear are examined. In the Indian Ocean, the WMC, which is active from December to March, presents an important vertical current shear (see zonal current inversion at 5° N in figure 2*c*). Figure 3*a* shows the nine tracks (nos. 4,7,9,10,13,16,17,20,21) that enter the WMC influence zone (above the 5° S parallel) during its period of activity. Therefore, these tracks cross both a region of high (region A) and of low (region B) vertical current shear during equivalent periods of the year. Figure 3*b* shows the impact of crossing these two regions on the relative error ε (equation (3.1)).

3.1 $$\varepsilon =\frac{\left\|{\hat{V}}_{s_{\left[\mathrm{TAD}\right]}}-{\hat{V}}_{s_{0}}\right\|}{\left\|{\hat{V}}_{s_{\left[\mathrm{TAD}\right]}}\right\|}$$

In region A, the average relative error *ε* is well above 10% (*ε* = 0.1), ranging from 0.5 ± 0.6 to 1.2 ± 1.3 while its value remains within the 10% limit in region B. These calculations expose that, in this case, approximating encountered currents with ${\hat{V_{c}}}_{0}$ is only acceptable below the 10° S parallel where vertical current shear is low. Above this line, the subsurface presence of the animal must be considered for swimming velocity calculations.

### 3.2. Removal of unrealistic swimming speed

**Figure 4.**
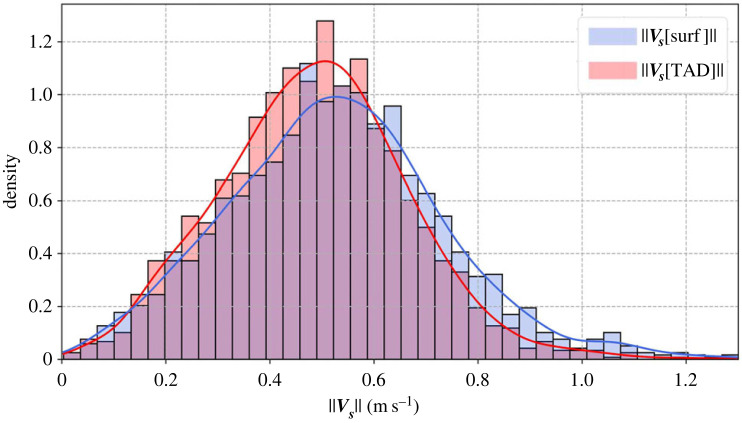
Global pelagic distribution of both swimming velocities for all group North individuals (*N* = 25).

Overall, using the TAD to calculate the swimming speed narrows the distribution of the norm around an average of 0.49 m s^−1^ compared with 0.53 m s^−1^ when only surface currents are considered (figure 4 and table 3). ${\hat{V}}_{s_{\left[\mathrm{TAD}\right]}}$ can remove 60% of the unrealistic daily swimming speed above 0.8 m s^−1^ in agreement with [50] who measured the swimming speed of similarly sized juvenile loggerheads (SCL approx. 60–80 cm). The remaining extreme values are probably due to residual model errors at the surface and in the subsurface layers, where the limited number of available observations reduces the benefit of data assimilation. Nevertheless, these results support the idea that these juvenile loggerheads migrate at a stable horizontal swimming speed, close to approximately 0.5 m s^−1^, which is better estimated using ${\hat{V_{s}}}_{\left[\mathrm{TAD}\right]}$, a swimming speed that accounts for TAD and vertical current shear.

**Table 3.** Statistics of the swimming velocity norm for ${\hat{V_{s}}}_{0}\;$ and ${\hat{V}}_{s_{\left[\mathrm{TAD}\right]}}$ in m s^−1^.

m s^−1^	$\left\|{\hat{V}}_{s_{0}}\right\|$	$\left\|{\hat{V}}_{s_{\left[\mathrm{TAD}\right]}}\right\|$
size	1821	1821
mean	0.53	0.49
s.d.	0.21	0.18
max	1.56	1.43

### 3.3. Piecewise constant heading swimming

**Figure 5.**
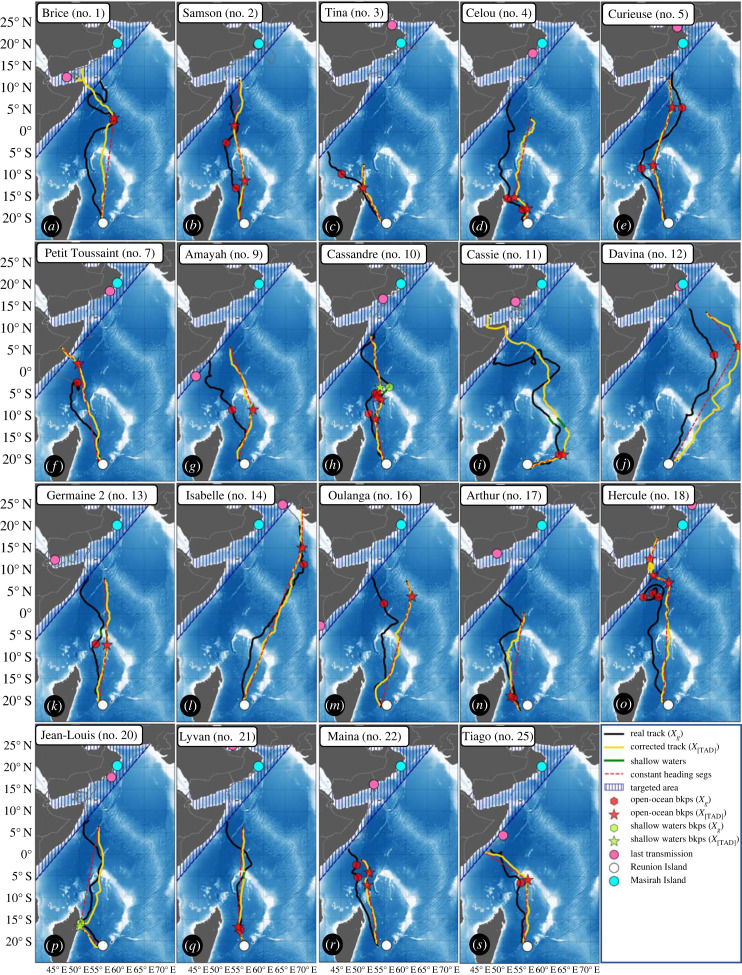
Real tracks *X_g_* (black) and corresponding corrected tracks *X*_[TAD]_ (yellow) atop the GEBCO bathymetry for the 19 selected individuals. Red stars (hexagons) indicate open-ocean breakpoints and green stars (hexagons) shallow water breakpoints on the current-corrected track *X*_[TAD]_ (real track *X_g_*). The red dashed lines are the constant heading segments where RMSE < 30°. Green segments are shallow water transects (bathymetry less than 200 m). Note that the corrected-track (yellow) is a fictious trajectory that only serves as visual proxy for the open-ocean navigation strategy, features of interest such as coastline or shallow water encounters are detected at matching positions on the real track.

The trajectories of the 19 individuals studied totalled 76 848 km and 1684 days in the open ocean. Of these, 66 584 km (87%) is accurately modelled (RMSE < 30°) by a piecewise constant heading model (table 4). For 13 of the 19 tracks, the entire open-ocean part of the trajectory was successfully approximated with only one or two breakpoints, revealing a navigation strategy based on long, clearly directed, swimming bouts with only a small number of heading correction events (figure 5 and electronic supplementary material, S3, table S3).

**Table 4.** Segmentation statistics for the 19 juvenile tracks for which *X*_[TAD]_ was calculated (see §2.4): distance travelled in the open ocean in km, distance travelled with constant heading (RMSE < 30°) in km, total number of breakpoints detected in relation to the number of breakpoints detected over shallow water (at the corresponding position on the real track where bathymetry < 200 m), angle of changes between successive valid segments (in degrees), frequency of breakpoints in km/bkps. For each track, the average of the straightness index and the average RMSE over segments of the modelled transect is calculated for tracks: *X*_[TAD]_, *X*_0_, *X_g_*.

Id	name	travelled distance open ocean	travelled distance constant heading	*Nbkps* (shallow water)	*Δθ* (°)	frequency of bkps (km/bkps)	average straightness index			average RMSE		
							TAD	surf	real	TAD	surf	real
1	Brice	5410	3102	1	X	3102	0.95	0.96	0.59	19.7	17.03	51.57
2	Samson	3521	3521	2	−12°, −17.5°	1761	0.98	0.97	0.95	11.68	13.81	18.3
3	Tina	2443	2443	1	−25.5°	2443	0.95	0.94	0.91	19.43	22.36	23.52
4	Celou	3936	3408	2	X	1704	0.91	0.86	0.86	24.76	30.75	32.97
5	Curieuse	4543	4543	2	−29.2°, 23.9°	2272	0.98	0.98	0.96	10.48	11.51	16.43
7	PetitToussaint	3165	3165	1	34°	3165	0.94	0.94	0.49	24.03	24.28	80.22
9	Amayah	3320	3320	1	36°	3320	0.94	0.94	0.82	20.79	20.32	37.56
10	Cassandre	4305	4205	4(1)	−26.1°, 1.7°	1051	0.95	0.89	0.86	17.19	27.32	30.16
11	Cassie	6572	912	1	X	912	0.94	0.91	0.81	19.5	26.45	33.36
12	Davina	4659	4659	1	70.3	4335	0.91	0.94	0.94	24.64	21.33	21.32
13	Germaine2	3673	3673	1	9.4	3673	0.95	0.87	0.87	17.61	29.42	28.04
14	Isabelle	5302	5302	1	21.2	5302	0.95	0.93	0.92	21.17	23.71	33.33
16	Oulanga	3892	3892	1	36.4	3892	0.94	0.87	0.9	19.74	29.15	23.77
17	Arthur	3552	3552	1	−41.4	3552	0.95	0.94	0.87	16.73	18.19	29.64
18	Hercule	5148	3915	3	X	1305	0.98	0.98	0.66	10.16	11.52	52.56
20	Jean-Louis	3942	3942	1(1)	−54.1	3942	0.93	0.92	0.88	22.23	22.89	29.58
21	Lyvan	3519	3519	1	−19.8	3519	0.97	0.93	0.89	13.52	21.92	27.06
22	Maina	2806	2371	2	X	1186	0.93	0.92	0.91	23.01	27.51	25.58
25	Tiago	3140	3140	1	59.6	3140	0.96	0.95	0.86	16.69	18.85	33.23
	**Mean**	4045	3487	1.5	0.3	2928	0.95	0.93	0.84	18.58	22.02	33.06
	**Sum**	76 848	66 584	28(2)

A total of 28 breakpoints were detected, giving an average distance of 2928 km between two breakpoints. Two of the breakpoints occur in shallow water (bathymetry less than 200 m), the rest occur in the deeper water. Furthermore, figure 5 shows that only one out of four trajectory segments over shallow water (green segments in figure 5*h,i,k,q*) was detected to have a course change (Cassandre no. 10), suggesting that heading changes are not systematically triggered by encounters with bathymetry features. Heading changes (Δ*θ*) between successive valid segments (*n* = 17) can be positive or negative (range: −54° to 70°) with a mean close to zero (= 0.3°) and a standard deviation of 42°. Such moderate heading changes occurring after nearly 3000 km long, relatively straight, trajectory segments reveal that juvenile loggerheads already have well-established navigational abilities and a clear navigation strategy at the time when they tackle their return trip towards their natal area.

The average straightness index over the identified segments for *X*_[TAD]_ is 0.95, not significantly higher than for the same segments of the *surface current-corrected track* (*X*_0_; 0.93; *t*-test, *p* = 0.07) but significantly higher for the *real track* (*X_g_*; 0.83; *t*-test, *p* < 0.001) (see table 4). The same observation is made for the average RMSE with 18.6°, 22.0° (*t*-test; *p* = 0.08), 33.1° (*t*-test; *p* < 0.001) respectively for *X*_[*TAD*]_, *X*_0_, *X_g_*. This confirms the potential of the current correction (*X*_[TAD]_ relatively to *X_g_*) to uncover these constant heading navigation segments. However, the benefit of using the three-dimensional over the two-dimensional approach is not evident from the statistical analyses here. Looking more specifically at the nine tracks that crossed the WMC, where a significant vertical shear can be found (nos. 4,7,9,10,13,16,17,20,21, see §3.1), the 5% statistical significance is now reached with *S* = 0.94 and RMSE = 18.9° using the TAD and *S* = 0.91 (*t*-test; *p* < 0.05) and RMSE = 24.0° (*t*-test; *p* < 0.05) using surface currents. This result supports the need to consider dive profiles to calculate the swimming velocity in oceanic regions where vertical shear is important.

## 4. Discussion

In the marine environment, it is crucial to retrieve the best possible encountered current velocity for the calculation of a realistic active swimming velocity. The present study shows that neglecting the subsurface presence of a marine animal can lead to a mis-estimation of the active motion and can consequently lead to a misinterpretation of the navigational skills and strategy.

### 4.1. When is it important to choose a three-dimensional approach for estimating swimming velocity of sea turtles?

The three-dimensional approach is required if the animal spends a significant amount of time in subsurface layers of the ocean where the current field differs significantly from the surface. Assessing whether such conditions exist can be difficult and requires knowledge of both the vertical structure of the region under study and the time-at-depth distribution of the population. Although recent improvements in both oceanic modelling [32,51,52] and biologging [53,54] are tending to open up this avenue, the two-dimensional approach is still convenient in some cases (computational cost, tag price, no TDRs data, old dataset, etc). In order to assess the validity of the two-dimensional approximation in the region studied, it is necessary to consider the maximum vertical shear likely to be encountered at depths where the animal spends a significant amount of time. For example, in the [−10°; 10°] equatorial band, an equatorial undercurrent flows eastward perennially in the Atlantic and Pacific [55] and seasonally in the Indian Ocean [56], causing vertical current shear between the upper and lower oceanic layers. Sea turtles occupy predominantly equatorial areas (60% of nesting sites are in the [−10°; 10°] band [57]. Although diving behaviour and water column use during pelagic migrations differ between species [58], the EUC and other deep currents influence their spatial distribution at different stages of their life cycle. In regions where the EUC is strong, considering the surface current as the sole drifting force will have two major effects: (i) it will artificially increase the swimming speed, and (ii) it will erroneously push the heading to the east, leading to difficulties in interpreting turtle's navigational strategy. This study argues that in certain regions of the world, measuring the TAD of marine animals is essential to properly analyse their navigational mechanisms.

### 4.2. On what navigational mechanisms do juvenile loggerhead sea turtles rely during their pre-reproductive migration?

The visual comparison of the *corrected* (*X*_[TAD]_) and *real tracks* (*X_g_*) (figure 5), as well as the result of the time-series heading segmentation shows that the removal of ocean currents tends to reveal piecewise straight-line swimming movements. It demonstrates the ability of these late-juvenile loggerheads to maintain a relatively constant heading over several hundred kilometres in the open ocean, probably relying on the sun and the magnetic field [44,59]. This ability, known as compass orientation, is only achievable by individuals with a true sense of compass [60] and has been documented as a plausible navigation strategy for several sea turtle species that target large continental areas [17,18,61]. This strategy appears compatible with the tracks of several individuals (figure 5*i,k,q,r*), that were able to reach the vicinity of the Omani or Somali coasts with limited course correction along the way (*Nbkps* = 1; |Δ*θ*| < 20°). For other individuals (figure 5*d,i,j,n,o,p,s*), clear course corrections (|Δ*θ*| > 40°) are detected en route either in the open ocean (red hexagons) or in shallow water (green hexagons). Regardless of whether course corrections were evident, it is likely that map information was used by all turtles, otherwise they would not have been able to set appropriate compass headings for the target region. These results are consistent with previous findings suggesting map and compass orientation in juvenile and adult sea turtles [11,19,62].

This work also tends to confirm that sea turtles do not achieve real-time compensation of the ocean current, but instead make intermittent course corrections, as the current correction removes part of the current variability by straightening the track. This can be clearly observed for tracks that include an encounter with an eddy (figure 5*a,i,o*); the straightening of the track when the current correction is applied indicates that individuals did not change course while being pushed back by these eddies. One possibility is that these individuals have only a coarse map sense [20] and are unable to make fine track corrections to counteract short-scale ocean currents. Another hypothesis is that, in order to limit energy expenditure during oceanic migrations, they do not attempt to counteract ocean currents as long as it does not prevent them from reaching their destination [63]. This idea is consistent with the stable swimming (mean = 0.48 m s^−1^, s.d. = 18 m s^−1^) speed during migration observed in this study.

In essence, our results support the idea of a map and compass navigation strategy for these juveniles in the open ocean, moving in a straight line and at a stable swimming speed, with intermittent course corrections along the way. This navigation strategy, which has been proposed for post-nesting green [19] or hawkbill [20] turtles, also appears to apply to juvenile loggerheads, outlining an inter-age and inter-specific navigation strategy for marine turtles.

### 4.3. Limitations

As presented here, the consideration of the TAD adds value to the estimation of the active motion, but this consideration depends on the accuracy of the modelled subsurface dynamics. Even though we know that the increasing number of subsurface observing systems (ARGO, Drifters, Glides, Moorings, Animal-born) has significantly improved our ability to model subsurface dynamics in recent years, there are still important uncertainties [64,65]. Furthermore, the operational forecast system PSY4 of Mercator Ocean, chosen here for its high resolution (1/12°) and its ability to provide data corresponding to the period of the tracks (until September 2022), does not offer the accuracy of a reanalysis model such as GLORYS12 (available until May 2021 at the time of the study) [32]. Other influences not modelled by either PSY4 or GLORYS12, such as Stokes drift, which is important in subtropical gyres, and tidal effects, which are important in coastal regions, would provide improvements in the retrieval of the active movement of marine animals. In terms of segmentation, the detection of breakpoints would benefit from combining the approach of [49] with the time series of heading segmentation using the common velocity field presented here to better assert the nature of each detected segment.

### 4.4. Perspectives

Here, the third dimension has been explored to provide better insight on the two-dimensional movement. In further studies, exploring the diving behaviour associated with this two-dimensional movement offers exciting perspectives to provide better understanding of sea turtle ecology during migrations. What is the diving routine of sea turtles during migrations? A regularity on the daily diving routine has been suggested here by making possible the reconstruction of a representative time-at-depth from 16% of time-series data (see electronic supplementary material, S1). How does this potential diving routine change upon encountering shallow waters? Is there a shallow water shift that is species-specific? Are the detected change points in the trajectory associated with transitions in the diving behaviour? It is clear that exploring the third dimension through the improvement of the animal-born technologies opens a new path to the understanding of sea turtle ecology as well as for other marine animals.

## Data Availability

The raw Argos locations, the daily interpolated files and the diving series are provided for the 32 investigated trajectories from the Zenodo digital repository: https://doi.org/10.5281/zenodo.8123489 [66]. Supplementary material is available online [67].
